# Concurrent transmission of multiple carbapenemases in a long-term acute-care hospital

**DOI:** 10.1017/ice.2023.231

**Published:** 2024-03

**Authors:** Danielle A. Rankin, Maroya Spalding Walters, Luz Caicedo, Paige Gable, Heather A. Moulton-Meissner, Allison Chan, Albert Burks, Kendra Edwards, Gillian McAllister, Alyssa Kent, Alison Laufer Halpin, Christina Moore, Tracy McLemore, Linda Thomas, Nychie Q. Dotson, Alvina K. Chu

**Affiliations:** 1 Florida Department of Health in Orange County, Orlando, Florida; 2 Bureau of Epidemiology, Florida Department of Health, Tallahassee, Florida; 3 Division of Healthcare Quality Promotion, Centers for Disease Control and Prevention, Atlanta, Georgia; 4 Division of Laboratory Services, Tennessee Department of Health, Nashville, Tennessee; 5 HCA Healthcare, Nashville, Tennessee

## Abstract

**Objective:**

We investigated concurrent outbreaks of *Pseudomonas aeruginosa* carrying *bla*_VIM_ (VIM-CRPA) and Enterobacterales carrying *bla*_KPC_ (KPC-CRE) at a long-term acute-care hospital (LTACH A).

**Methods:**

We defined an incident case as the first detection of *bla*_KPC_ or *bla*_VIM_ from a patient’s clinical cultures or colonization screening test. We reviewed medical records and performed infection control assessments, colonization screening, environmental sampling, and molecular characterization of carbapenemase-producing organisms from clinical and environmental sources by pulsed-field gel electrophoresis (PFGE) and whole-genome sequencing.

**Results:**

From July 2017 to December 2018, 76 incident cases were identified from 69 case patients: 51 had *bla*_KPC,_ 11 had *bla*_VIM,_ and 7 had *bla*_VIM_ and *bla*_KPC_. Also, *bla*_KPC_ were identified from 7 Enterobacterales, and all *bla*_VIM_ were *P. aeruginosa*. We observed gaps in hand hygiene, and we recovered KPC-CRE and VIM-CRPA from drains and toilets. We identified 4 KPC alleles and 2 VIM alleles; 2 KPC alleles were located on plasmids that were identified across multiple Enterobacterales and in both clinical and environmental isolates.

**Conclusions:**

Our response to a single patient colonized with VIM-CRPA and KPC-CRE identified concurrent CPO outbreaks at LTACH A. Epidemiologic and genomic investigations indicated that the observed diversity was due to a combination of multiple introductions of VIM-CRPA and KPC-CRE and to the transfer of carbapenemase genes across different bacteria species and strains. Improved infection control, including interventions that minimized potential spread from wastewater premise plumbing, stopped transmission.

Among the underlying mechanisms of bacterial carbapenem resistance, carbapenemases are of significant public health concern. Carbapenemases are frequently encoded on mobile genetic elements (eg, plasmids) that often contain additional resistance determinants, can be transferred between bacterial taxa,^1^ and are associated with rapid increases in carbapenem resistance.^2–4^ In the United States, nearly 35% of carbapenem-resistant Enterobacterales (CRE) harbor a carbapenemase, with *Klebsiella pneumoniae* carbapenemase (KPC) most commonly identified.^5^ Carbapenemases are less commonly the mechanism of carbapenem resistance in *Pseudomonas aeruginosa*; ∼2% of carbapenem-resistant *Pseudomonas aeruginosa* (CRPA) harbor a carbapenemase, most frequently the Verona-integron-encoded metallo-β-lactamase (VIM).^4,6,7^ Carbapenemase-producing organisms (CPOs) can cause outbreaks in healthcare facilities resulting in infections with limited treatment options.^2,8,9^

Patients most susceptible to acquiring CPOs have complex medical needs.^10,11^ Most patients with CPOs are asymptomatically colonized, presenting challenges for identification and initiation of transmission-based precautions.^7^ CPO transmission may occur via transient hand carriage by healthcare personnel or via contaminated shared medical equipment. Additionally, a growing body of literature describes CPO transmission from healthcare facility wastewater plumbing to patients.^3,12,13^

On July 5, 2017, the Florida Department of Health (FDOH) was notified of 2 carbapenemase-producing organisms, VIM-producing *Pseudomonas aeruginosa* and KPC-producing *Klebsiella pneumoniae,* in specimens from a patient of long-term acute-care hospital-A (LTACH A) on admission to a local acute-care hospital. In response, the FDOH conducted an onsite infection control assessment at LTACH A that identified significant gaps in hand hygiene and transmission-based precautions adherence, raising concern for transmission. In July 2017, a facility-wide point-prevalence survey (PPS) of 36 patients identified 4 patients with KPC-producing CRE and 3 patients with VIM-producing CRPA. Here, we describe epidemiologic and laboratory investigations to control transmission.

## Methods

### Setting

LTACH A is a freestanding facility with a 6-bed intensive care unit (ICU) and a progressive care unit that expanded from 40 to 50 private rooms in January 2018.

### Case definitions and case finding

We defined a case as the detection of *bla*_VIM_ or *bla*_KPC_ in a clinical isolate or screening specimen from a patient admitted to LTACH A for ≥1 night between July 13, 2017, and December 18, 2018. An incident case was the first identification of *bla*_VIM_ or *bla*_KPC_; case patients could have 2 incident cases (1 *bla*_VIM_ and 1 *bla*_KPC_). Incident cases were considered to have been acquired in LTACH A if they were identified from a patient without a history of colonization or infection with that carbapenemase and with ≥1 negative screening result at least 1 week before incident specimen collection. Cases considered present on admission had specimens collected within 3 days of admission to LTACH A.

In July 2017, we requested the commercial laboratory of LTACH A to submit carbapenem-resistant organisms identified in clinical specimens to the FDOH Bureau of Public Health Laboratory (BPHL) for carbapenem resistance mechanism testing. In August 2017, we initiated admission and discharge screening and biweekly facility-wide point-prevalence surveys (PPSs) to detect carbapenemase genes. Human subjects advisors in Florida reviewed the investigation activities and determined that they constituted public health response. This research was exempt from human subjects review by the Centers for Disease Control and Prevention (CDC) and was conducted consistent with applicable federal law and CDC policy [45 C.F.R. part 46.102(I)(2)].

### Case investigation

We completed medical record reviews for incident cases using a standard abstraction form to collect patient demographics, past medical history, underlying medical conditions, discharge status, presence of indwelling device(s), and antibiotic administration at the time of or in the 14 days before incident specimen collection. We calculated slopes of newly acquired cases and CPO prevalence by fitting a linear regression line in 2-week intervals (based on PPSs) over the outbreak duration using R Studio version 1.2.1335 software (R Foundation for Statistical Computing, Vienna, Austria).

### Cohort study

We conducted a retrospective cohort study to assess risk factors associated with *bla*_VIM_ and/or *bla*_KPC_ acquisition during the initial months of the outbreak. All patients admitted to LTACH A from July 5 to December 7, 2017, with ≥2 colonization screenings performed were included. Information regarding maintenance of medical devices, hemodialysis procedures, enteral feedings, respiratory therapy, speech, occupational, and physical therapy as well as peripherally inserted central catheter (PICC) insertion and line maintenance were obtained from procedure log books. Medical records were not abstracted for noncases; thus, we were unable to select comorbid conditions as a confounder in our regression models. Mortality data were collected through Florida’s electronic death registry.^14^ Univariable analysis was conducted using the Welch unequal variance *t* test for continuous variables and the Pearson χ^2^ test for categorical variables. Confounders were identified using prior knowledge. A multivariable logistic regression adjusting for age, sex, length of stay, and ICU admission was conducted to estimate the relative risks (RRs) and 95% confidence intervals (CIs) for acquisition of *bla*_VIM_ and *bla*_KPC_ during hospitalization at LTACH A. Statistical tests based on a 2-tailed probability and significance level of α = 5% were conducted using Stata IC version 16.0 software (Statacorp LLC, College Station, TX).

### Infection control observations and interventions

Scheduled and unannounced infection control assessments with observations of practice were conducted using the CDC Infection Control Assessment and Response (ICAR) Tool for Acute Care Hospitals (www.cdc.gov/hai/prevent/infection-control-assessment-tools.html). We audited adherence to the World Health Organization Five Moments for Hand Hygiene^15^ and recorded hand hygiene (HH) and personal protective equipment (PPE) observations via the iScrub Lite mobile phone application (version 1.5.3, 2018, SwipeSense, Chicago, IL). We also observed environmental cleaning, respiratory care, antibiotic compounding, and device reprocessing.

### Laboratory investigation

Carbapenem-resistant Enterobacterales and *P. aeruginosa* from clinical cultures were forwarded to the CDC and the BPHL for carbapenem resistance mechanism testing. Colonization screenings were conducted by testing rectal swabs for carbapenemase genes using the Cepheid Xpert CarbaR (Cepheid, Sunnyvale, CA).^16^ when carbapenemase genes were detected, a swab was cultured to recover carbapenem-resistant organisms (Supplementary File 1 online).

### Environmental sampling

Environmental samples were collected from sink drains, splash zone surfaces, and mobile equipment (Supplementary Table 1 online). Environmental samples underwent broth enrichment and plating onto selective media agar to screen for suspect isolates.

**Table 1.** Characteristics of Case Patients with Carbapenemase-Producing Organisms, by Carbapenemase Gene Detected at Long-Term Acute-Care Hospital-A, Florida, July 2017–December 2018^a^

Characteristics	All CPOs (n=61), No. (%)^b^	*bla*_VIM_, only (n=9), No. (%)^b^	*bla*_VIM_ and *bla*_KPC_ (n=6), No. (%)^b,c^	*bla*_KPC_, only (n=46), No. (%)^b,d^	*P* Value^e^
Sex, male	34 (56)	7 (78)	2 (33)	25 (54)	.220
Age, median y (IQR)	66 (56–73)	60 (42–72)	67 (64–72)	66 (56–73)	.790
LOS median d (IQR)	48 (28–77)	41 (28–57)	82 (42–89)	47 (27–73)	.857
ICU stay before incident specimen	22 (36)	3 (33)	4 (67)	15 (33)	.259
Duration to incident specimen, median d (IQR)	34 (20–52)	20 (14–27)	33 (21–48)	36 (24–57)	.135
**Specimen type of incident case**					
Rectal screening	53 (87)	7 (78)	4 (67)	42 (91)	.166
Clinical isolate	8 (13)^f^	2 (18)	2 (33)	4 (8)	
**Epidemiologic classification**					
Met criteria for LTACH-A acquired^a^	40 (66)	2 (22)	5 (83)	33 (72)	.011^h^
Unable to determine if LTACH-A acquired^g^	21 (34)	7 (78)	1 (17)	13 (28)	
Death, <90 d incident specimen	20 (33)	4 (44)	4 (67)	12 (26)	.103^i^
LOS, from first-positive to death, median d (IQR)	33 (16–60)	41 (34–58)	20 (8–45)	28 (16–65)	.614
Charlson comorbidity score, median (IQR)	3 (1–5)^j^	3 (0–5)	6 (5–8)	3 (2–4)^k^	.014^h,i^
No Charlson comorbidities^k^	5 (8)	3 (33)	0	2 (4)	.006
**5 Most common Charlson comorbidities**					
Diabetes	31 (65)^j^	3 (33)	6 (100)	22 (67)^k^	.027^h,i^
Diabetes with complications	13 (27)^j^	1 (11)	5 (83)	7 (21)^k^	.003^h,i^
Congestive heart failure	16 (33)^j^	4 (44)	4 (67)	8 (24)^k^	.094
Chronic pulmonary disease	13 (27)^j^	1 (11)	3 (50)	9 (27)^k^	.252
Renal disease	12 (25)^j^	3 (33)	2 (33)	7 (21)^k^	.668
**Current device(s) present or 14 days before incident specimen**					
BiPAP/CPAP	5 (9)^l^	1 (11)	0 (0)	4 (9)^m^	.718
Feeding tube	55 (93)^l^	9 (100)	6 (100)	39 (91)^m^	.473
Mechanical ventilation	31 (53)^l^	5 (56)	6 (100)	20 (47)^m^	.048^h^
PICC line	28 (48)^l^	4 (44)	4 (67)	20 (47)^m^	.632
Tracheostomy	42 (72)^l^	7 (78)	6 (100)	29 (67)^m^	.229
Urinary catheter	18 (31)^l^	5 (56)	2 (33)	11 (25)^m^	.191
**≥**3 devices	43 (62)^l^	8 (73)	6 (86)	29 (57)^m^	.248
**Special care services**					
Hemodialysis	22 (38)^l^	4 (44)	3 (50)	15 (35)^m^	.704
Decubitus ulcers	12 (27)^n^	3 (38)^o^	2 (40)^p^	7 (22)^q^	0.519
**Antibiotic therapy, 14 d before incident specimen**					
Meropenem	16 (36)^r^	4 (50)^n^	3 (60)^o^	9 (29)^s^	.277
Vancomycin	23 (52)^r^	4 (50)^n^	4 (80)^o^	15 (48)^s^	.418
Cefepime	9 (20)^r^	3 (38)^n^	1 (20)^o^	5 (16)^s^	.409

Note. VIM, Verona-integron-encoded metallo-β-lactamase; KPC, *Klebsiella pneumoniae* carbapenemase; LOS, length of stay; ICU, intensive care unit; BiPAP/CPAP, bilevel/continuous positive airway pressure; PICC, peripherally inserted central catheter. *P* values were calculated using the Pearson χ^2^ test for categorical variables and the Welch unequal variance *t* test for continuous variables.

a Excludes 8 case patients who had CPO identified from admission screening.

b Units unless otherwise specified.

c For patients with both *bla*_VIM_ and *bla*_KPC_, 5 of 6 had both carbapenemase genes identified on the same date (n=3) or within 7 days (n=2); date-specific analyses were performed using the date of specimen collection for the first incident case.

d One of the 46 KPC cases was identified as KPC-CRPA, the remainder were KPC-CRE.

e Pairwise comparison between *bla*_KPC_ and *bla*_VIM_ was <.05.

f Clinical specimen sources include sputum (n=2), urine (n=4), and wounds (n=2).

g Unable to conclusively assign time-point of CPO acquisition of no admission screening established before our first PPS and for those case-patients identified before when admission screening was implemented.

h Pairwise comparison between *bla*_VIM_ vs. *bla*_VIM_ and *bla*_KPC_ was <.05.

i Pairwise comparison between *bla*_KPC_ vs. *bla*_VIM_ and *bla*_KPC_ was <.05.

j n=48.

k No Charlson comorbidity index includes patients who may have had other comorbid conditions but did not have conditions included in the Charlson comorbitiy index.

l n=58.

m n=43.

n n=45.

o n=8.

p n=5.

q n=32.

r n=44.

s n=31.

### Molecular characterization

Pulsed-field gel electrophoresis (PFGE) was performed on clinical and environmental isolates. A subset of isolates was selected for short-read whole-genome sequencing (WGS) based on epidemiological findings and representativeness of isolates in distinct PFGE clusters. The isolates that underwent short-read WGS also underwent long-read WGS to better resolve plasmid structures (Supplementary File 2 online).

## Results

### Outbreak overview

From July 13, 2017, to December 18, 2018, 76 incident cases were identified from 69 case patients: 11 had *bla*_VIM_, 51 had *bla*_KPC,_ and 7 had *bla*_VIM_ and *bla*_KPC_. All *bla*_VIM_ were identified in *P. aeruginosa*, and *bla*_KPC_ was identified in 19 *Klebsiella pneumoniae*, 7 *Citrobacter freundii*, 5 *Enterobacter cloacae* complex, 1 *Klebsiella oxytoca*, 1 *Serratia marcescens*, 1 *Providencia rettgeri*, 1 *Providencia stuartii*, and 1 *Citrobacter farmeri*. Also, 5 patients had multiple organisms harboring *bla*_KPC_, and an organism was not recovered from 18 screening tests in which *bla*_KPC_ was identified.

In total, 8 case patients (2 *bla*_VIM_, 5 *bla*_KPC_, 1 *bla*_KPC_ and *bla*_VIM_) were identified from admission screens; 53 case patients (7 *bla*_VIM_, 42 *bla*_KPC_, 4 *bla*_KPC_ and *bla*_VIM_) were identified from PPS or discharge screens; and 8 case patients (2 *bla*_VIM_, 4 *bla*_KPC_, 2 *bla*_KPC_ and *bla*_VIM_) were identified from clinical cultures.

### Incident cases and prevalence

**Figure 1.**
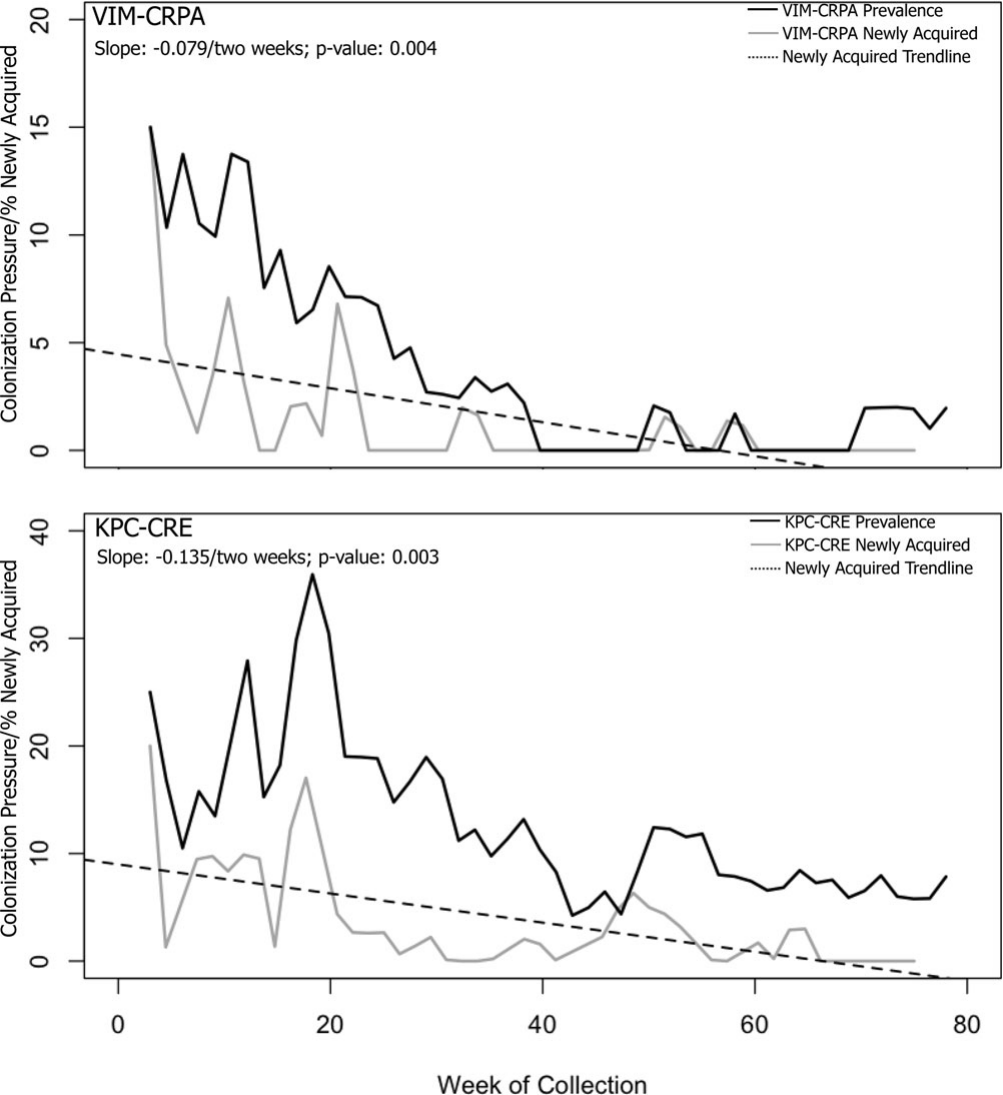
Prevalence and new acquisitions of carbapenemase-producing organisms detected through colonization screening at long-term acute-care hospital A, Florida, July 2017–December 2018. Prevalence (ie, colonization pressure) is the total number of cases currently hospitalized/census. Newly acquired indicates the percentage of patients with incident *bla*_VIM_ and *bla*_KPC_ among all screened patients. Note. VIM-CRPA, Verona-integron-encoded metallo-beta-lactamase–producing carbapenem-resistant *Pseudomonas aeruginosa*; KPC-CRE, *Klebsiella pneumoniae* carbapenemase–producing carbapenem-resistant Enterobacteriaceae.

From July 2017 to December 2018, a gradual decrease in incidence of *bla*_VIM_ (slope, −0.079 every 2 weeks; *P =* .004) and *bla*_KPC_ (slope, −0.135 every 2 weeks; *P =* .003) was observed (Fig. 1). Declines were sharpest during the first 6 months (July 2017–January 2018: *bla*_VIM_ slope, −0.220 every 2 weeks, *P =* .021 and *bla*_KPC_ slope, −0.353 every 2 weeks; *P =* .018). The investigation closed on December 18, 2018, after no newly acquired cases were identified in LTACH A for 2 consecutive months.

### Clinical characteristics and risk factors of case patients not identified on admission to LTACH A

Patient characteristics and risk factors for the 61 case patients (88%) who did not have a CPO identified on admission are described in Table 1. The median age was 66 years (IQR, 56–73 years). Demographic risk factors were similar among patients with *bla*_VIM_, *bla*_KPC_, and both *bla*_VIM_ and *bla*_KPC_. Case patients with both *bla*_VIM_ and *bla*_KPC_ had higher median Charlson scores and were more likely to have diabetes than those with *bla*_VIM_ alone (median score, 6 vs 3; *P* = .033; diabetes, 100% vs 33%, respectively; *P ≤* .05) or *bla*_KPC_ alone (median score, 6 vs 3; *P* = .048; diabetes, 100% vs 67%, respectively; *P ≤* .05).

### Cohort study

From July 5 to December 7, 2017, 146 patients were hospitalized at LTACH A, of whom 98 (67%) met our cohort study inclusion criteria. Among the 98 patients in the cohort, the 22 patients with CPO acquired at LTACH A had similar demographics to the 76 who did not acquire a CPO. Healthcare risk factors differed in that a greater proportion of case patients had PICC lines (68% vs 40%; *P* = .017) and ≥3 indwelling devices (77% vs 41%; *P* = .003) (Table 2).

**Table 2.** Demographic and Clinical Characteristics of Patients with and without Hospital-Acquired Carbapenemase-Producing Organisms (CPOs) During Initial Months of an Outbreak, Long-Term Acute-Care Hospital A (LTACH A), Florida, July 13–December 7, 2017

Characteristic	No CPO (n=76), No. (%)^a^	Any CPO (n=22), No. (%)^a^	*P* Value	*bla*_VIM^b^_ (n=6), No. (%)^a^	*P* Value	*bla*_KPC^b^_ (n=20), No. (%)^a^	*P* Value
Sex, male	37 (48)	12 (55)	.628	3 (50)	.979	10 (50)	.959
Age, median y (IQR)	67 (59–73)	66 (55–72)	.874	71 (65–80)	.581	66 (59–71)	.957
LOS, median (IQR)	32 (23–50)	43 (34–58)	.190	46 (39–62)	.373	43 (35–58)	.121
Any ICU stay at LTACH A	14 (18)	6 (27)	.364	1 (17)	.814	6 (30)	.233
Duration to first positive or last negative swab, median d (IQR)	30 (21–42)	29 (23–41)	.392	22 (20–32)	.097	31 (24–41)	.813
Negative colonization swab(s), median (IQR)	3 (2–3)	1 (1–2)	<.001	2 (1–2)	.002	1 (1–2)	<.001
**Location at time of first positive or last negative swab**							
Unit A	31 (41)	8 (36)	.814	3 (50)	.691	6 (30)	.510
Unit B	37 (49)	11 (50)		1 (17)		3 (15)	
ICU	31 (41)	8 (36)		2 (33)		11 (55)	
Oral vancomycin	4 (5)	4 (18)	.051	2 (33)	.020	4 (20)	.030
**Device(s) present at time of or before incident specimen collection**							
BiPAP/CPAP	10 (13)	2 (9)	.608	0 (0)	.342	2 (10)	.731
Feeding tube	61 (80)	21 (95)	.090	6 (100)	.264	19 (95)	.124
Hemodialysis vascular access device	19 (25)	9 (41)	.146	2 (33)	.790	9 (45)	.068
Mechanical ventilation	34 (45)	13 (59)	.235	5 (83)	.073	12 (60)	.227
PICC line	30 (40)	15 (68)	.017	4 (67)	.293	14 (70)	.015
Tracheostomy	34 (45)	14 (64)	.118	6 (100)	.010	12 (60)	.269
≥3 indwelling devices^c^	31 (41)	17 (77)	.003	6 (100)	.010	15 (75)	.009

Note. VIM, Verona-integron-encoded metallo-β-lactamase; KPC, *Klebsiella pneumoniae* carbapenemase; LOS, length of stay; ICU, intensive care unit; BiPAP/CPAP, bilevel/continuous positive airway pressure; PICC, peripherally inserted central catheter. *P* values were calculated using the Pearson χ^2^ test for categorical variables and the Welch unequal variance *t* test for continuous variables.

a Units unless otherwise specified.

b 4 patients had both VIM and KPC detected; referent group for VIM is no VIM detection; referent group for KPC is no KPC detection.

c Device cutoff was determined a priori.

Patients with a feeding tube or ≥3 indwelling medical devices had an increased risk of acquiring *bla*_KPC_ (aRR, 1.18; 95% CI, 1.01–1.39; aRR, 1.21 95% CI, 1.02–1.43, respectively) and *bla*_VIM_ (aRR, 1.07; 95% CI, 1.02–1.14; aRR, 1.14; 95% CI, 1.03–1.26) relative to patients without a feeding tube or with <3 indwelling medical devices (Table 3). The risk of acquiring *bla*_VIM_ increased with the presence of a tracheostomy and decreased with receipt of bilevel positive airway pressure (BiPAP) or continuous positive airway pressure (CPAP).

**Table 3.** Association of Medical Exposures and Acquisition of Carbapenemase-Producing Organisms During Initial Months of an Outbreak, by Carbapenemase Gene Detected, Long-Term Acute-Care Hospital A (LTACHA), Florida, July 13–December 7, 2017

Medical Exposures	*bla*_VIM_			*bla*_KPC_		
	Adjusted RR^a^	95% CI	*P* Value	Adjusted RR^a^	95% CI	*P* Value
Oral vancomycin	1.22	0.92–1.63	.170	1.37	0.98–1.93	.066
Feeding tube	1.07	1.02–1.14	.022	1.18	1.01–1.39	.041
BiPAP/CPAP	0.94	0.89–0.98	.015	0.96	0.76–1.22	.744
Hemodialysis	1.02	0.93–1.12	.695	1.14	0.93–1.40	.195
Mechanical ventilation	1.09	0.97–1.23	.163	1.05	0.89–1.25	.519
PICC line	1.05	0.93–1.18	.437	1.21	1.02–1.45	.030
Speech therapy	1.12	0.97–1.29	.127	1.08	0.64–1.85	.752
Tracheostomy	1.13	1.03–1.24	.010	1.07	0.91–1.25	.408
≥ 3 indwelling devices^b^	1.14	1.03–1.26	.014	1.21	1.02–1.43	.025

Note. VIM, Verona-integron-encoded metallo-β-lactamase; KPC, *Klebsiella pneumoniae* carbapenemase; LOS, length of stay; ICU, intensive care unit; BiPAP/CPAP, bilevel/continuous positive airway pressure; PICC, peripherally inserted central catheter. *P* values were based on a 2-tailed probability and a significance level set at α < .05.

a All models were adjusted for age, sex, length of stay, and intensive care unit stay.

b Device cutoff was determined a priori; the referent group for medical exposures was the absence of the device and the referent group for ≥3 devices was 0–2 devices. Device history was collected through procedure logs provided by LTACH-A on a monthly basis.

### Infection control assessments and interventions

**Figure 2.**
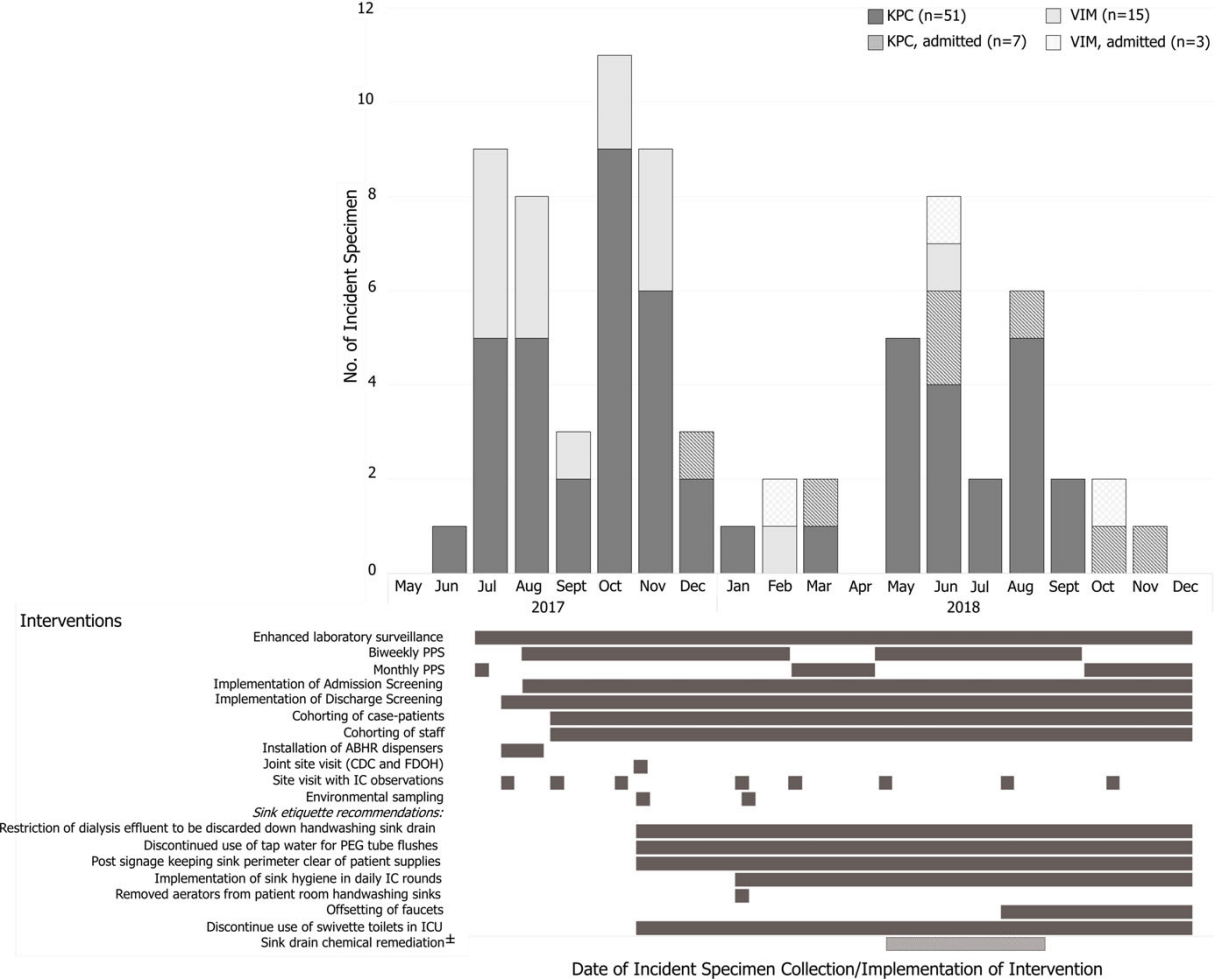
Epidemic curve and timing of infection control interventions to control new acquisitions of carbapenemase-producing organisms detected at long-term acute-care hospital (LTACH) A, Florida, July 2017–December 2018. “±” denotes action taken by LTACH A, but not recommended by public health officials. “KPC, admitted” represents patients identified with KPC at the time of admission. “VIM, admitted” represents patients identified with VIM at the time of admission. The incident specimen is the specimen that yielded the patient’s first identified organism and mechanism combination. Note. CDC, Centers for Disease Control and Prevention, Division of Healthcare Quality and Promotion; FDOH, Florida Department of Health; IC, infection control; PPS, point-prevalence screenings.

Beginning in July, we conducted 5 announced and 8 unannounced infection control assessments. At the initial assessment, we observed appropriate hand hygiene (HH) in 61% of opportunities and glove and gown use in 61% and 67% of opportunities, respectively. Access to alcohol-based hand rub (ABHR) and personal protective equipment (PPE) was limited. Recommended interventions included increasing HH and PPE audits and access to ABHR and PPE, placing case patients in cohorts by CPO status to different wings with dedicated patient-care staff, and scheduling case-patient specialized care appointments (eg, hemodialysis and physical therapy) and daily room cleaning after patients without known CPOs. However, CPO acquisitions remained high through October 2017 (Fig. 2 and Supplementary File 3 online).

A follow-up assessment in November 2017 identified continued gaps in adherence to HH and contact precautions and multiple practices with potential to transmit CPOs from wastewater plumbing to patients, including storing medical supplies such as syringes used to flush enteral feeding tubes in the sink splash zone or above the swivette toilet, discarding nutritive materials in the hand washing sink, cleaning from the sink basin to the countertop, and compounding oral vancomycin in close proximity to a hand washing sink.

The FDOH provided HH and PPE training with return demonstration to ∼225 LTACH healthcare personnel; overall adherence at the next assessment was 90% for HH and 92% for both glove and gown use and was sustained in all 5 subsequent assessments over a 10-month period. Over several months, LTACH A implemented recommendations to mitigate spread from plumbing by assessing for patient care items in sink splash zones during daily infection control rounds. LTACH A also discontinued use of sinks for liquid waste disposal, adding reminder signage near sinks, and offsetting faucets from the drain (Fig. 2). Although not a public health recommendation, LTACH A treated drains with bleach for 4 months beginning in May 2018; this practice coincided with decreased attention to other interventions intended to reduce transmission from sink drains and correlated with a resurgence of *bla*_KPC_ cases.

### Environmental investigation

In November 2017 and January 2018, 91 environmental samples were collected from high-touch surfaces, medical equipment, sink drains in patient rooms, the pharmacy, medicine preparation rooms, and patient toilets. In total, 5 different Enterobacterales harboring *bla*_KPC_ and 2 *Pseudomonas* spp harboring *bla*_VIM_ were recovered from high-touch surfaces, sinks, and wastewater plumbing (Table 4).

**Table 4.** Carbapenemase-Producing Organisms Detected from Environmental Samples Collected at Long-Term Acute-Care Hospital-A, Florida, November 2017 and January 2018^a^

Location/Site	Mechanism	Organism
ICU swivette toilet	*bla*_VIM-61_	*Pseudomonas putida*
Patient room handwashing sink drain	*bla*_VIM-2_, *bla*_VIM-61_	*P. aeruginosa*
Patient room handwashing sink surface area	*bla*_VIM-2_, *bla*_VIM-48_	*P. aeruginosa*
ICU patient room handwashing sink drain	*bla*_VIM_^a^	*P. putida*
ICU swivette toilet	*bla*_KPC-3_	*Enterobacter asburiae*
Patient room handwashing sink drain	*bla*_KPC-3_	*E. cloacae* *E. kobei* *E. asburiae*
Patient room handwashing sink drain	*bla*_KPC-3_	*E. cloacae*
Patient room handwashing sink drain	*bla*_KPC-3_	*Citrobacter freundii*
Patient room handwashing sink surface area	*bla*_KPC-4_	*E.cloacae*
ICU patient room handwashing sink drain	*bla*_KPC-3_	*E. asburiae*
Medication dispensing room sink drain	*bla*_KPC-2_	*Klebsiella pneumoniae*
Pharmacy sink drain	*bla*_KPC-3_	*Serratia marcescens*

Note. ICU, intensive care unit.

a All environmental samples collected from long-term acute care hospital-A are provided in Supplementary Table 1 (online). In total, 91 environmental samples were collected. Whole-genome sequencing was not performed.

### Molecular characterization of clinical and environmental isolates

**Fig. 3.**
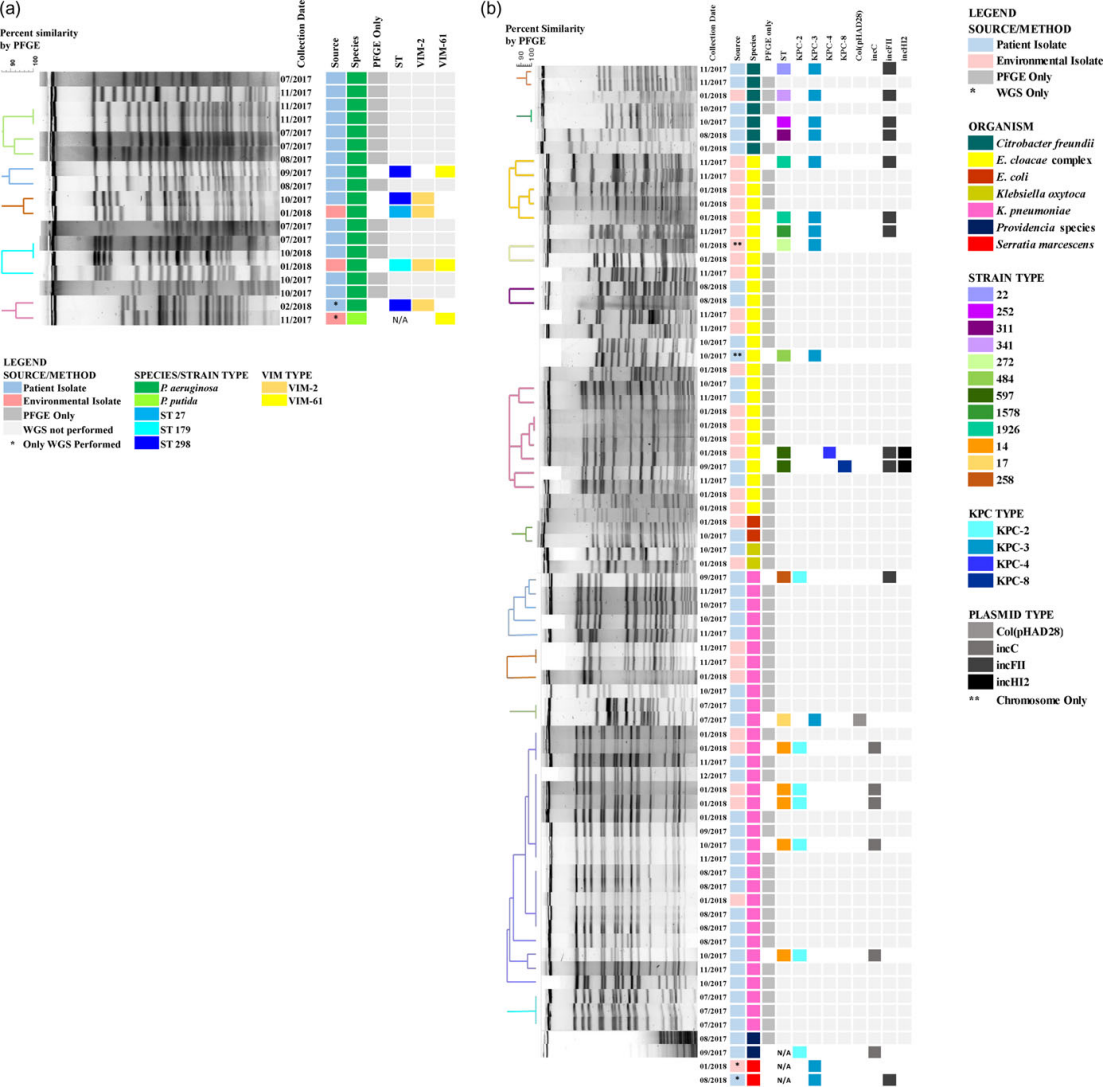
Pulsed-field gel electrophoresis and whole-genome sequencing results for clinical and environmental isolates with (A) VIM-CRPA and (B) KPC-CRE detected at long-term acute-care hospital (LTACH) A, Florida, July 2017–December 2018.

A PFGE dendrogram annotated with metadata including the sequence type (ST) and carbapenemase alleles for the subset of representative isolates that underwent WGS are shown in Figure 3. Among 21 KPC-producing CRE that underwent WGS, 4 KPC alleles were identified: 7 *bla*_KPC-2_, 12 *bla*_KPC-3_, 1 *bla*_KPC-4_, and 1 *bla*_KPC-8_. Also, *bla*_KPC-2_ and *bla*_KPC-3_ were identified in different Enterobacterales and in both clinical and environmental isolates. Furthermore, 6 isolates with *bla*_KPC-2_ including 5 *K. pneumoniae* ST14 corresponding to the largest PFGE cluster identified, and 1 *Providencia stuartii*, harbored the gene on an IncC plasmid. Also, 12 isolates with *bla*_KPC-3_ represented 4 species: 5 *E. cloacae* complex, 4 *C. freundii*, 2 *S. marcescens*, and 1 *K. pneumoniae*. Among the 11 with long-read sequence data available, *bla*_KPC-3_ was identified on a Col (pHAD28) plasmid in 1 *K. pneumoniae* ST17 and on an IncFII plasmid in 4 *C. freundii*, 3 *E. cloacae* complex, and 1 *S. marcescens*, and on the chromosome in 2 *E. cloacae* (Fig. 3A).

No single dominant PFGE cluster was observed for VIM-CRPA (Fig. 3B). Among 6 VIM *Pseudomonas* isolates from 3 patients and 3 environmental sources that underwent WGS, 2 alleles were identified: 4 *bla*_VIM-2_ and 3 *bla*_VIM-61_ and were indentified in both clinical and environmental isolates. *bla*_VIM-61_ is a novel allele closely related to *bla*_VIM-7_ and was identified in a patient’s *P. aeruginosa* ST298 isolate that was part of a PFGE cluster and in 2 environmental isolates.

## Discussion

Our public health response to the identification of a patient colonized with VIM-CRPA and KPC-CRE revealed large, concurrent CPO outbreaks in an LTACH. Epidemiologic and laboratory evidence suggest that the observed diversity in organisms and mechanisms is explained by ongoing CPO importation and carbapenemase gene transfer across different species and strains. Several factors contributed to transmission. We controlled the outbreak by improving core infection control practices, intervening on spread from sink drains, and initiating admission, PPS, and discharge screening.

LTACHs in the United States play a vital role in managing critically ill patients requiring long hospitalizations. LTACHs can serve as amplifiers of multidrug-resistant organism (MDRO) transmission due to the combination of (1) the complex patient population they serve, (2) challenges with implementing infection control practices aimed at preventing transmission,^17,18^ and (3) patient sharing with other healthcare facilities. In LTACH A, unrecognized importation of CPOs, combined with inadequate training and support for core infection control practices, likely contributed to spread of CPOs among patients and to the healthcare environment, creating reservoirs of resistant bacteria. Improved infection control practices andcoupled with enhanced detection of CPOs upon admission helped prevent later introductions from wider dissemination. Although the admission prevalence was relatively low (2%), LTACH A is regionally influential through patient sharing networks^19^ and has continued admission screening in partnership with public health due to the perceived value of proactively identifying patients with CPOs. Outbreaks at LTACHs, as well as intensive interventions to prevent MDRO transmission in this setting, may have meaningful impacts on increasing or decreasing, respectively, regional MDRO spread.^20–24^ Thus, these sustained efforts at LTACH-A may provide considerable benefits to the broader region.

Most case patient isolates with *bla*_KPC-2_ belonged to large PFGE clusters of *K. pneumoniae* that were identified early in the investigation (September 2017–January 2018), when adherence to core infection control practices was poor. Isolates harboring *bla*_KPC-3_ corresponded to the 2 largest PFGE clusters of *E. cloacae* and 3 other Enterobacterales and were identified for the duration of the outbreak. Although the diversity of PFGE patterns and periodic identification of case patients on admission could have led to the conclusion that KPC cases were due to multiple introductions followed by small clusters of transmission, added resolution from WGS suggests horizontal transfer of plasmids among species may have contributed to some of the observed diversity. As short- and long-read WGS become increasingly available, their integration into public health responses may improve identification of plasmid outbreaks.

CPO outbreaks attributed to hospital wastewater plumbing have been increasingly reported, with sink drains being the most recognized reservoir.^25–28^ Wastewater plumbing is readily contaminated with CPOs during patient care; the biofilm omnipresent in plumbing structures provides a fertile environment for plasmid exchange.^2,3^ Although recovery of CPOs from wastewater plumbing does not indicate directionality of spread,^26^ several factors increase the plausibility of CPO transmission from wastewater plumbing to patients at LTACH A. These include cleaning practices that disseminated contaminants from the sink basin to surrounding area, recovery of CPOs from the sink splash zone where supplies were stored, and control of transmission following improved adherence to sink hygiene. Additionally, risk factors identified in the cohort study, the presence of a feeding tube and receipt of oral vancomycin, were linked to observed sink hygiene gaps: storage of syringes for feeding tube flushes within the sink and swivette toilet splash zones and compounding of oral vancomycin adjacent to a pharmacy sink drain from which KPC-CRE was recovered. Although wastewater plumbing is hypothesized to have been the source of many transmissions, person-to-person CPO transmission also contributed, as demonstrated by some geographic clustering of cases (data not shown) and epidemiologic links to specialty care services (eg, hemodialysis), particularly early in the outbreak when adherence to hand hygiene and PPE use was low.

The index case was the first VIM-CRPA reported in Florida.^13^ LTACH-A VIM-CRPA isolates, however, showed surprising diversity in alleles and strain types, indicating that VIM-CRPA may be more common in central Florida than previously recognized. Supporting this finding, ∼1% of patients screened at admission carried *bla*_VIM_. Furthermore, some patient and environmental isolates harbored an allele, *bla*_VIM-61_, that appears to be unique to central Florida and has been identified in patients without epidemiologic linkages to LTACH-A. These findings emphasize the value of admission screening and collaboration with other healthcare facilities and public health partners to prevent further spread of CPOs in the central Florida region.

Our investigation had several limitations. First, we used paper procedure logs to identify exposure risk factors in the cohort study, and we were unable to evaluate some potential confounders, such as comorbid conditions and antibiotic receipt for noncases. For many cases, we were unable to conclusively determine whether they were acquired at LTACH A because they were identified before implementation of admission screening. Finally, we selected a subset of isolates representing varied PFGE patterns and specimen sources for WGS to infer isolate relatedness; however, it is possible that isolates with related PFGE patterns could harbor different carbapenemase alleles or plasmid markers. Isolates with plasmids sharing the same replicon and carbapenemase allele could represent plasmids from different sources and may indicate evolution of plasmid genes during the outbreak or unique plasmids introduced to LTACH A.

Through epidemiologic and molecular investigations, we identified concurrent outbreaks of carbapenemase-producing organisms. The primary reservoirs and modes of transmission may have varied among the different alleles and organisms and at different stages of the outbreak. This investigation illustrates how sustained public health and healthcare facility collaboration can control spread of emerging resistance in high-acuity postacute care facilities.

## Supplementary material

For supplementary material accompanying this paper visit https://doi.org/10.1017/ice.2023.231. click here to view supplementary material
