# Supplementary material for: Concurrent transmission of multiple carbapenemases in a long-term acute-care hospital
Source: Infect Control Hosp Epidemiol. 2024 Jan 10;45(3):292–301. doi: 10.1017/ice.2023.231 (PMC10933503; doi:10.1017/ice.2023.231)
Supplement: Supplementary file 1 [file S0899823X23002313sup001.docx]

**Online Supplemental Files Table of Content**

Supplemental File 1. Laboratory Phenotypic Testing Methods for Carbapenem Resistance and Carbapenemase Prtion Methods

Supplemental File 3. Additional Details on Infection Control Assessments and Interventions at LTACH-A

Supplemental Table 1. Environmental Samples Collected from Long-Term Acute Care Hospital A, Florida

**Supplemental File 1. Laboratory Phenotypic Testing Methods for Carbapenem Resistance and Carbapenemase Production**

Broth microdilution was used to determine the minimal inhibitory concentrations of bacterial isolates according to standards established by the Clinical and Laboratory Standards Institute**^1^**, and the modified Carbapenem Inactivation Method (mCIM) for detection of carbapenemase-producing bacteria based on the 2015 CIM procedure**^2^**. Using the Applied Biosystems’ Fast 7500 Real-Time PCR System (Applied Biosystems, Inc., Foster City, CA), isolates demonstrating carbapenemase-production underwent RT-PCR testing for the detection of five carbapenemase genes: *bla*_VIM,_ *bla*_IMP,_ *bla*_NDM,_ *bla*_OXA-48-type,_ and *bla*_KPC_.”

1. Clinical and Laboratory Standards Institute (CLSI) documents M7, M45, and M100
2. Van der Zwaluw, K., A. de Haan, G.N. Pluister, H.J. Bootsma, A.J. de Neeling, and L.M. Schouls. (2015). The Carbapenem Inactivation Method (CIM), a Simple and Low-Cost Alternative for the Carba NP Test to Assess Phenotypic Carbapenemase Activity in Gram-Negative Rods. *PLoS ONE 10(3)*: e0123690.

**Supplemental File 2. Molecular Characterization Methods**

All suspect isolates were identified by MALDI-TOF using the Bruker and the CDC MicrobeNet databases (<https://microbenet.cdc.gov/>). DNA was extracted directly from broth enrichment and pure culture isolates using the Promega Maxwell 16 Cell Low Elution Volume (LEV) DNA Purification Kit and Maxwell 16 MDx Instrument (Madison, WI). Detection of carbapenemase genes was performed using a CDC Multiplex RT-PCR assay ([Laboratory Resources | HAI | CDC](https://www.cdc.gov/hai/settings/lab/lab_settings.html)).

CDC performed pulse-field gel electrophoresis (PFGE) and/or whole genome sequencing (WGS) on *bla*_VIM_- and *bla*_KPC_-harboring environmental and patient isolates to determine relatedness. PFGE typing was performed as described by CDC PulseNet ([Pulsed-field Gel Electrophoresis (PFGE) | PulseNet Methods| PulseNet | CDC](https://www.cdc.gov/pulsenet/pathogens/pfge.html)), and genetic relatedness of isolates was analyzed by BioNumerics software (Applied Maths, Belgium).

WGS was performed on a subset of environmental and patient isolates at CDC and by the Tennessee Division of Laboratory Services. We selected a subset of isolates for short-read WGS that represented a diversity of PFGE patterns and both environmental isolates and isolates from patients with suspected CPO acquisition at LTACH A. Environmental and clinical isolates that represented different MLST and organisms were selected for long-read WGS. At CDC, DNA was extracted from pure culture isolates (Promega Maxwell 16 Cell Low Elution Volume DNA Purification Kit and Maxwell 16 MDx Instrument (Madison, WI)) and subsequently sheared (Covaris ME220 Focused-ultrasonicator (Woburn, MA)). Indexed libraries were prepared (Tecan Ovation Ultralow System V2 Kit (San Carlos, CA); PerkinElmer Zephyr G3 NGS Workstation (Waltham, MA)) and analyzed (Standard Sensitivity NGS Fragment Analysis Kit and Agilent Fragment Analyzer System (Santa Clara, CA)). Sequencing was performed using the MiSeq Reagent V2 Kit and Illumina MiSeq System (San Diego, CA) generating 2x250 paired-end reads. Illumina reads were assembled and annotated with Quaisar-H, a description of tools is available at: <https://github.com/DHQP/QuAISAR_singularity>. For long-read sequencing, DNA was extracted using the Promega Wizard® Genomic DNA Purification Kit (Madison, WI) and quantified (Qubit dsDNA BR Assay Kit and NanoDrop 2000/2000c Spectrophotometer). Long-read sequence data were generated on a PacBio RS II platform (Pacific Biosciences. Menlo Park, CA) at CDC’s core sequencing facility. PacBio reads were assembled using either HGAP (v3) or Canu then polished with Quiver and circularized with Circulator to generate high quality, complete genome and plasmid assemblies.

**Supplemental File 3. Additional Details on Infection Control Assessments and Interventions at LTACH-A**

***CPO Cohorting***

LTACH-A is comprised of multiple wings of private rooms. We cohorted case-patients by CPO status to different wings with dedicated patient-care staff, and scheduled case-patient specialized care appointments (e.g., hemodialysis, physical therapy) and daily room cleans after patients without known CPOs to reduce cross-contamination. Case-patients with both *bla_KPC_* and *bla_VIM_* were cohorted with the case-patients with *bla_VIM._* In the intensive care unit, although geographic separation was not possible, LTACH-A dedicated patient care staff and scheduled specialty care appointments and daily room cleans last on the unit.

**Supplemental Table 1. Environmental Samples Collected from Long-Term Acute Care Hospital A, Florida**

| **Site/Location** | **Date of Collection** | **Specimen Type** |
| --- | --- | --- |
| Room A Handsink, 1^st^ catch | 11/8/2017 | Bulk |
| Room A Handsink, post-flush | 11/8/2017 | Bulk |
| Room A Bedside table | 11/8/2017 | Swab |
| Room A IV pole (dynascope) | 11/8/2017 | Swab |
| Room A Dialysis connector | 11/8/2017 | Swab |
| Room A Faucet | 11/8/2017 | Swab |
| Room A Sink drain | 11/8/2017 | Swab |
| EVS cart, composite swab | 11/8/2017 | Swab |
| Scope cabinet, bottom area | 11/8/2017 | Swab |
| Olympus cart, composite swab | 11/8/2017 | Swab |
| Keyboard | 11/8/2017 | Swab |
| Scope sample | 11/8/2017 | Swab |
| Faucet, endoscope cleaning | 11/8/2017 | Swab |
| Gasket of Steris system IE | 11/8/2017 | Swab |
| Connector gasket (feed ports) of Steris system IE | 11/8/2017 | Swab |
| Alcohol cleaning tube | 11/8/2017 | Swab |
| EVS cart | 11/8/2017 | Swab |
| Nurses cart | 11/8/2017 | Swab |
| Room B Handsink, 1st catch | 11/8/2017 | Bulk |
| Room B Handsink, post-flush | 11/8/2017 | Bulk |
| Room B Hopper | 11/8/2017 | Swab |
| Room B Counter | 11/8/2017 | Swab |
| Room B Drain | 11/8/2017 | Swab |
| Room B Faucet | 11/8/2017 | Swab |
| Room C Handsink, 1st catch | 11/8/2017 | Bulk |
| Room C Handsink, post-flush | 11/8/2017 | Bulk |
| Room C Drain | 11/8/2017 | Swab |
| Room C Faucet | 11/8/2017 | Swab |
| Room C IV pole (stethoscope) | 11/8/2017 | Swab |
| Vent port 03 (not used) | 11/8/2017 | Swab |
| Vent port 11 (in use) | 11/8/2017 | Swab |
| Vent port 20 (not used) | 11/8/2017 | Swab |
| Vent port 23 (not used | 11/8/2017 | Swab |
| 1st POU sink, post flush, cold only | 12/5/2017 | Bulk |
| Exterior hose bib | 12/5/2017 | Bulk |
| Room D Patient room hand sink | 12/5/2017 | Aerator |
| Room E Patient room hand sink | 12/5/2017 | Aerator |
| Pharmacy Sink Drain | 1/24/2018 | Swab |
| Pharmacy Handwash Sink | 1/24/2018 | Sponge Stick |
| Dialysis Machine F102 | 1/24/2018 | Sponge Stick |
| Dialysis Machine FT125 | 1/24/2018 | Sponge Stick |
| Dialysis Machine F73 | 1/24/2018 | Sponge Stick |
| Dialysis Machine F78 | 1/24/2018 | Sponge Stick |
| Dialysis Room Sink Drain | 1/24/2018 | Swab |
| Dialysis Room Sink Faucet Y- Left | 1/24/2018 | Swab |
| Dialysis Room Sink Faucet Y-Right | 1/24/2018 | Swab |
| Room F Faucet | 1/24/2018 | Swab |
| Room G Active Patient Faucet | 1/24/2018 | Swab |
| Room G Active Patient Sink Area | 1/24/2018 | Sponge Stick |
| Room G Active Patient Sink Drain | 1/24/2018 | Swab |
| Room G Active Patient Bed Rail | 1/24/2018 | Sponge Stick |
| Transport Chair | 1/24/2018 | Sponge Stick |
| Room G Active Patient Vent Surface | 1/24/2018 | Sponge Stick |
| Room H Active Patient Faucet | 1/24/2018 | Swab |
| Room H Active Patient Sink Area | 1/24/2018 | Swab |
| Room H Active Patient Sink Drain | 1/24/2018 | Swab |
| Room H Active Patient Bed Rail | 1/24/2018 | Sponge Stick |
| Room H Active Patient Bedside Table | 1/24/2018 | Sponge Stick |
| Room I Active Patient Faucet | 1/24/2018 | Swab |
| Room I Active Patient Sink Area | 1/24/2018 | Swab |
| Room I Active Patient Drain | 1/24/2018 | Swab |
| Room I Active Patient Bed Rail | 1/24/2018 | Sponge Stick |
| Room I Active Patient Bedside Table | 1/24/2018 | Sponge Stick |
| Room I Active Patient Call Light | 1/24/2018 | Sponge Stick |
| Room J Active Patient Faucet | 1/24/2018 | Swab |
| Room J Active Patient Sink Area | 1/24/2018 | Swab |
| Room J Active Patient Drain | 1/24/2018 | Swab |
| Room J Active Patient Bed Rail | 1/24/2018 | Sponge Stick |
| Room J Active Patient Bedside Table | 1/24/2018 | Sponge Stick |
| Room J Active Patient IV Pole | 1/24/2018 | Sponge Stick |
| Pharmacy Hood Surface Sample | 1/24/2018 | Sponge Stick |
| Pharmacy Work Surface Area | 1/24/2018 | Sponge Stick |
| Pharmacy sink counter surface area | 1/24/2018 | Sponge Stick |
| South Nutrition Ice Machine Ice Spout | 1/24/2018 | Swab |
| South Nutrition Ice Machine Water Spout | 1/24/2018 | Swab |
| South Nutrition Ice Machine Drain | 1/24/2018 | Swab |
| South Nutrition Sink Drain | 1/24/2018 | Swab |
| South Nutrition Counter | 1/24/2018 | Sponge Stick |
| Room F Active Patient Sink Drain | 1/24/2018 | Swab |
| Room F Active Patient Call Light | 1/24/2018 | Sponge Stick |
| Room F Active Patient Bed Rail | 1/24/2018 | Sponge Stick |
| South Side Medication Room Sink Drain | 1/24/2018 | Swab |
| South Side Medication Room Sink Faucet | 1/24/2018 | Swab |
| South Medication Room Dispenser | 1/24/2018 | Sponge Stick |
| Kitchen hand wash Sink Drain | 1/24/2018 | Swab |
| Kitchen hand wash Sink Faucet | 1/24/2018 | Swab |
| South Nutrition Ice Machine | 1/24/2018 | Swab |
